# The Western Hospital, Torquay

**Published:** 1916-01-29

**Authors:** W. F. Manley


*To the Editor of* The Hospital.

### The Western Hospital, Torquay.

*To the Editor of* The Hospital.

Sir,—Referring to your Note on the work of this hospital, on p. 338 of the January 15 issue, the explanation of the apparently small amount of dividends is that the dividends on the £5,299 legacy under the will of the late Mr. Lavers have to be shown as "Subscription X.L.," and not lumped into one. Still, I can see that it would be desirable for public information that the item "X.L." should be explained in future.—Yours faithfully, W. F. Manley.

January 21, 1916.

